# *Trioza turouguei* sp. nov. (Hemiptera, Psylloidea, Triozidae), a new psyllid species from Taiwan inducing pea-shaped stem galls on *Cinnamomum osmophloeum* (Lauraceae), with notes on its galling biology

**DOI:** 10.3897/zookeys.958.52977

**Published:** 2020-08-11

**Authors:** Gene-Sheng Tung, Yi-Chang Liao, Daniel Burckhardt, Man-Miao Yang

**Affiliations:** 1 Division of Botanical Garden, Taiwan Forestry Research Institute, Council of Agriculture, Executive Yuan, 53, Nanhai Rd., Taipei 100, Taiwan Taiwan Forestry Research Institute Taipei Taiwan; 2 Department of Entomology, National Chung Hsing University, 145, Xinda Rd., Taichung 402, Taiwan National Chung Hsing University Taichung Taiwan; 3 Naturhistorisches Museum, Augustinergasse 2, 4001 Basel, Switzerland Naturhistorisches Museum Basel Switzerland

**Keywords:** Asia, jumping plant lice, Oriental region, phenology, *
Siphonaleyrodes
*, Sternorrhyncha, taxonomy

## Abstract

*Trioza turouguei***sp. nov.**, a new species of jumping plant lice (Hemiptera, Triozidae) from Taiwan, is described and illustrated based on adults and immatures. The latter induce pea-shaped galls on the stems of *Cinnamomum osmophloeum* Kaneh. (Lauraceae). The gall phenology of the new species is described. A list of species of Triozidae associated with *Cinnamomum* in the Old World is provided. The following nomenclatorial acts are proposed: *Trioza inflata* Li, 1992 = *Trioza xiangicamphorae* Li, 1992, **syn. nov.**; *Siphonaleyrodes formosanus* Takahashi, 1932, **stat. rev**., is removed from synonymy with *Trioza cinnamomi* (Boselli, 1931).

## Introduction

Jumping plant lice or psyllids (Hemiptera, Sternorrhyncha, Psylloidea) are phloem-feeding insects that are highly host specific, especially during the immature stages (Hodkinson 1974). The superfamily is comprised of approximately 4,000 species in more than 200 genera worldwide (Li 2011; Burckhardt and Ouvrard 2012; Ouvrard 2020). Closely related psyllid species tend to develop on closely related plant species (Burckhardt and Basset 2000; Percy et al. 2004; Ouvrard et al. 2015; Burckhardt and Queiroz 2020). As other phytophagous insects, many psyllids are gall inducers, particularly those of the families Triozidae, Phacopteronidae, and Calophyidae (Burckhardt 2005; Malenovský et al. 2007; Yang and Raman 2007). Psyllid galls are characterized by a very specific morphology, formation site, and restriction to a single or a few related plant species (Hodkinson 1984; Burckhardt 2005).

The first studies on the psyllid fauna of Taiwan are from foreign researchers made during the first half of the 20^th^ century (Kuwayama 1908, 1910, 1931; Enderlein 1914). Half a century later, Yang (1984) published the first comprehensive monograph, which was subsequently supplemented and expanded (Fang and Yang 1986; Yang et al. 1986; Lauterer et al. 1988; Fang 1990; Yang et al. 2004, 2009, 2013; Liao et al. 2016; Liao and Yang 2018; Cho et al. 2020). According to these studies, more than half of the Taiwanese psyllid species are gall inducers and several of these are associated with the Lauraceae (Yang et al. 2006), a family of Magnoliids, an early branch in the angiosperm tree. Hollis and Martin (1997) compiled a list of the known psyllids associated with Lauraceae and recorded ten species developing on the lauraceous genus *Cinnamomum* in the Oriental realm.

*Cinnamomum osmophloeum* Kaneh. is a tree species endemic to Taiwan, growing at low elevations around the island. The tree species has some economic potential for its essential oils in the leaves (Chang et al. 2001), which are similar to those found in the bark of Indonesian cassia (*Cinnamomum burmanni* (Nees & T. Nees) Blume) with anti-bacterial, carminative, and anti-fungal properties. On the stems of *C. osmophloeum*, pea-shaped closed galls were found, in the field as well as on herbarium specimens in Taiwan, which are induced by an undescribed psyllid species. According to Hodkinson (1984, 2009), stem galls induced by psyllids are relatively rare compared to the much more common leaf galls. For this reason, also little is known about the phenology of stem galls.

Here, we formally name the species on *C. osmophloeum* as *Trioza turouguei* sp. nov., describe its adults and immatures, discuss its relationships to other psyllids developing on *Cinnamomum*, and provide information on the life cycle and gall phenology.

## Materials and methods

Psyllids were collected by sweeping and directly searching on *Cinnamomum osmophloeum*. The material is dry mounted or preserved in 70% and 99% ethanol. Some specimens were cleared in 15% potassium hydroxide and examined in orange oil or glycerol or permanently mounted in Canada balsam on a slide. Information on galls was taken in the field and from herbarium specimens.

Specimens from following institutions were examined: Entomological Museum, China Agricultural University, Beijing, China (**CAUB**); National Chung Hsing University, Taichung, Taiwan (**NCHU**); Naturhistorisches Museum, Basel, Switzerland (**NHMB**); National Museum of Natural Science, Taichung, Taiwan (**NMNS**); Herbarium of School of Forestry and Resource Conservation, National Taiwan University, Taipei, Taiwan (**NTUF**); Herbarium of National Taiwan University, Taipei, Taiwan (**TAI**); Herbarium of Taiwan Forest Research Institute, Taipei, Taiwan (**TAIF**).

Photographs of most morphological characters were taken with a compound microscope (Leica DM 750) equipped with a digital camera (Canon EOS 600D). Images of the forewings of adults were taken with a stereomicroscope (Leica MZ 125) equipped with a digital camera (Olympus EP-1). The photographs were montaged using focus stacking software (Helicon Focus, Helicon Soft). The morphological terminology follows White and Hodkinson (1982), Ossiannilsson (1992), Hollis (2004) and Yang et al. (2013).

The life cycle and gall phenology were observed at the Huisun Experimental Forest Station (24°05'24"N, 121°02'03"E; 660–370 m a.s.l.) from January to December 1996. We selected eight trees of *C. osmophloeum* to record the phenology of the plants and the galls induced by *T. turouguei* sp. nov. The terminology of gall development follows Lalonde and Shorthouse (1984) and Rohfritsch (1992). The stage of immatures inside the gall was checked by dissection of the gall.

## Taxonomy

### Trioza turouguei sp. nov.

Taxon classificationAnimalia

FFF7F165-CA99-5D79-A761-36A7C85B1DB3

http://zoobank.org/A5C30F1F-9539-40E2-A2E0-5ECDEEBECC01

**Figure 1.**
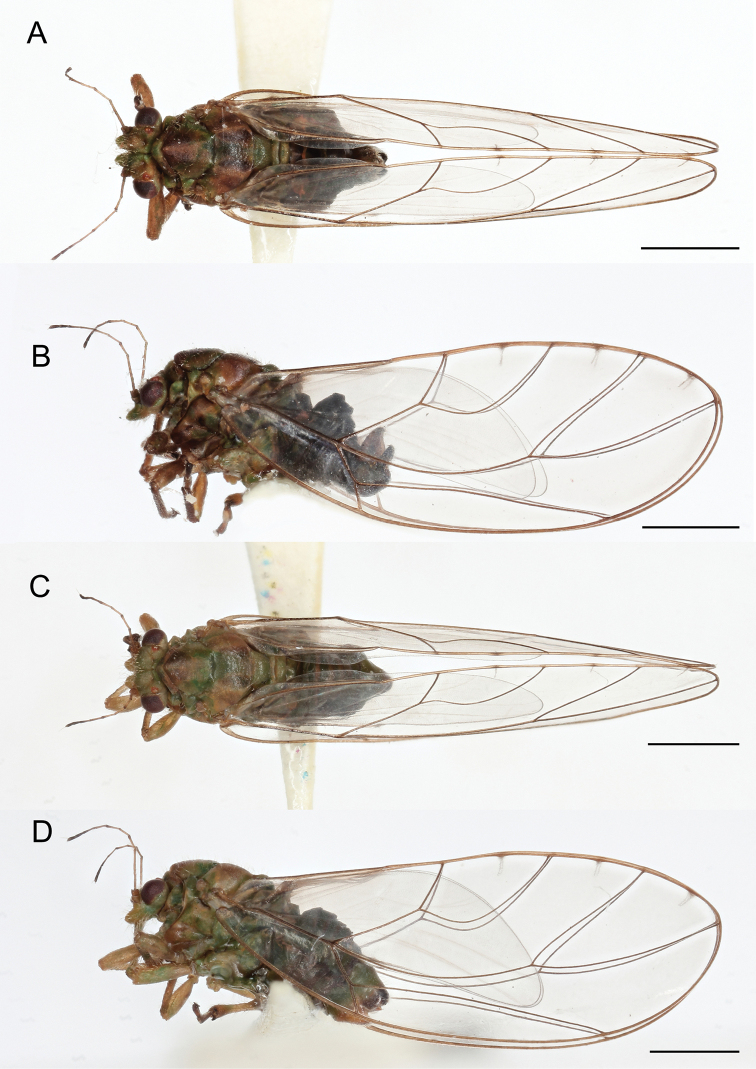
Adults of *Trioza turouguei* sp. nov. **A** male, dorsal view **B** male, lateral view **C** female, dorsal view **D** female, lateral view. Scale bars: 1 mm.

**Figure 2.**
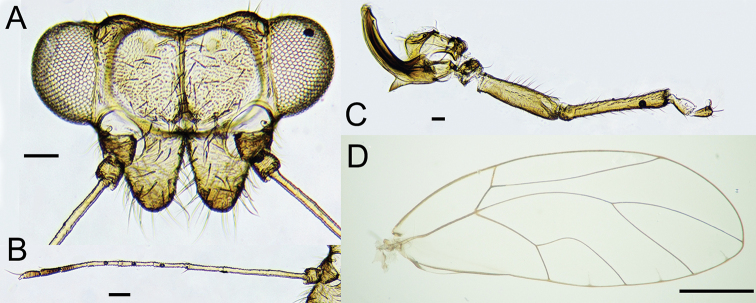
*Trioza turouguei* sp. nov. **A** head **B** antenna **C** hind leg **D** forewing. Scale bars: 0.1 mm (**A, B, C**); 1 mm (**D**).

**Figure 3.**
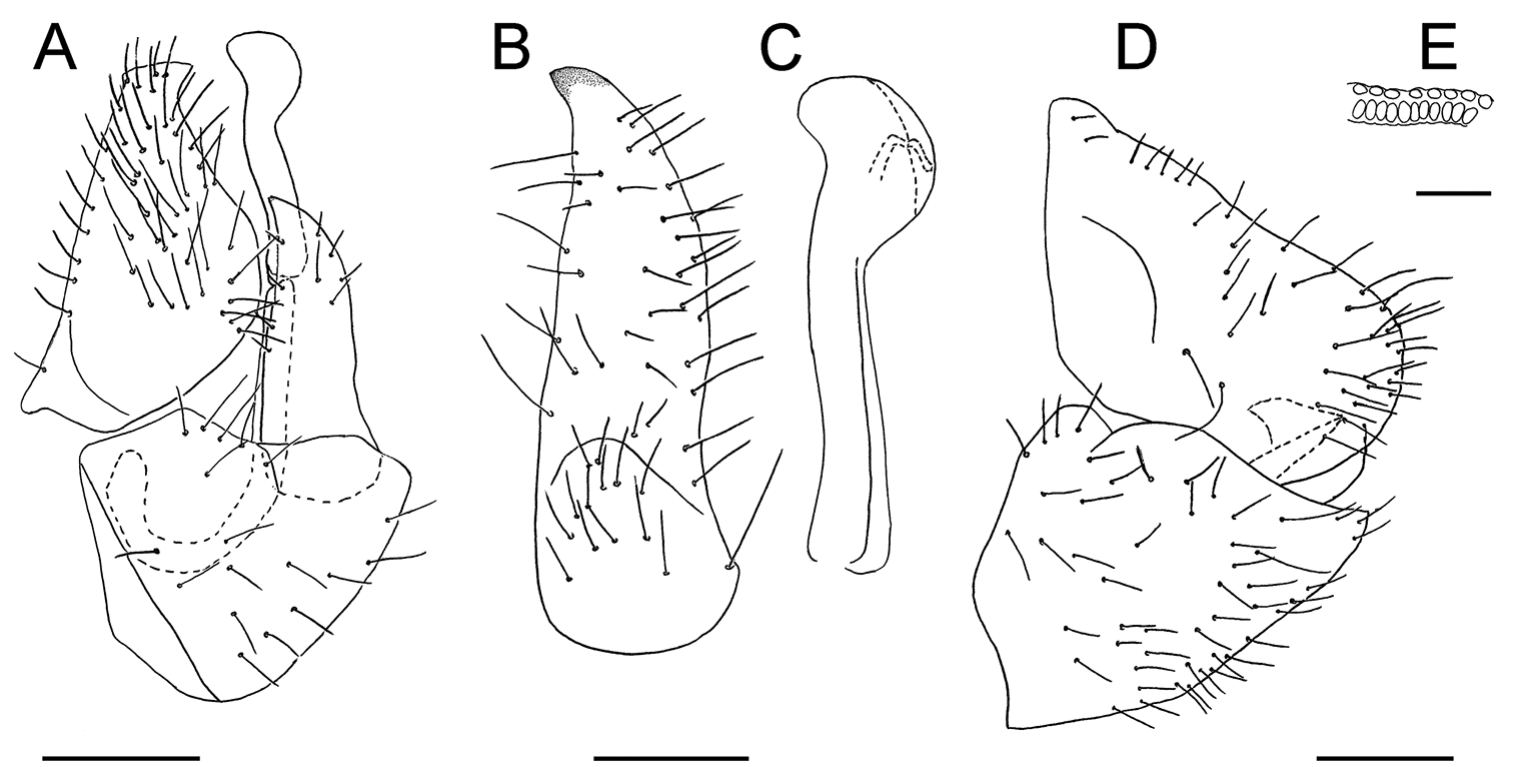
Terminalia of *Trioza turouguei* sp. nov. in lateral view **A** male terminalia **B** paramere, inner surface **C** distal portion of aedeagus **D** female terminalia **E** detail of female circumanal ring. Scale bars: 0.2 mm (**A, D, E**); 0.1 mm (**B, C**).

**Figure 4.**
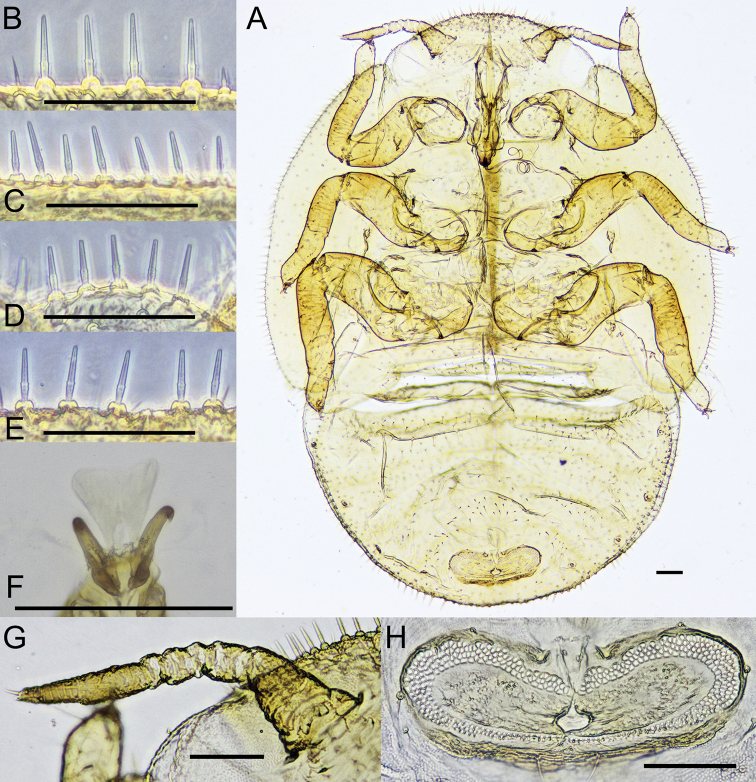
Fifth instar immature of *Trioza turouguei* sp. nov. **A** habitus **B** marginal sectasetae of head **C** marginal sectasetae of forewing pad **D** marginal sectasetae of hindwing pad **E** marginal sectasetae of caudal plate **F** tarsal arolium **G** antenna **H** circumanal ring. Scale bars: 0.1 mm.

**Figure 5.**
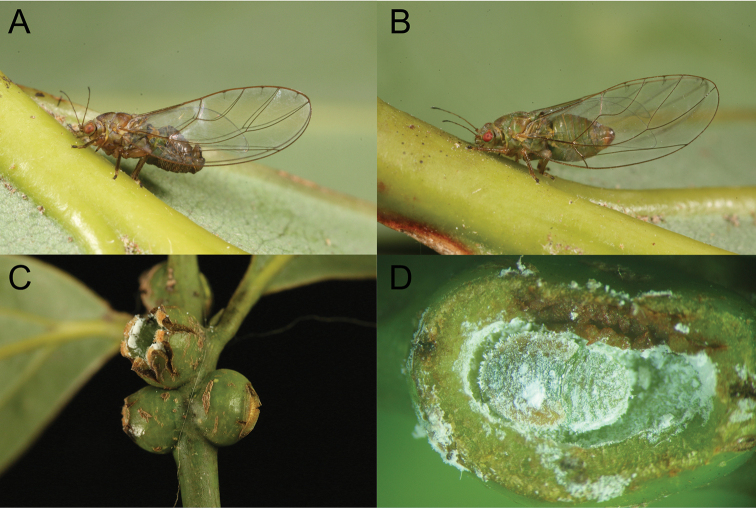
*Trioza turouguei* sp. nov. on its host plant, *Cinnamomum osmophloeum* Kanehira **A** male **B** female **C** pea-like galls on stem **D** fifth instar immature in a gall.

Figs 1 , 2 , 3 , 4 , 5

#### Type material.

***Holotype***: Taiwan • ♂; Taichung City, Shalien Lane; 24°11'20"N, 120°55'06"E; 20 Dec. 2018; Y. C. Liao leg.; *Cinnamomum osmophloeum*; NCHU, dry mounted. ***Paratypes***: Taiwan • 15 ♂, 17 ♀, 13 immatures; same data as for holotype • 1 ♀; same data as for holotype but 31 Jan. 2018 • 13 ♂, 23 ♀, 4 immatures, 1 skin; Taichung City, Upper Kukuan; 27 Jan. 2006; G. S. Tung leg.; *Cinnamomum osmophloeum* • 1 ♂, 1 ♀, 6 immatures; Nantou Co., Hui-Sun Forest Station; 24°05'24"N, 121°02'03"E; 17 Jan. 1996; G. S. Tung and M. M. Yang leg.; *Cinnamomum osmophloeum* • 6 immatures; same locality as for preceding; 24 Dec. 1996; G. S. Tung leg.; *Cinnamomum osmophloeum*. Paratypes in NCHU, NHMB, NMNS, dry and slide mounted or stored in ethanol.

#### Other material examined

(not included in type series). Galls on herbarium specimens of *Cinnamomum osmophloeum*, Taiwan: • Nantou Co., Meiyuunshan; 8 Oct. 1935; TAI 049104 • Nantou Co., Shuishe; 1 Mar. 1918; TAIF 107581 • Taichung City, Tungmaoshan; 5 Apr. 1984; TAI 194343 • Taichung City, Pahsienshang; 6 Dec. 1985; TAIF 123377, 123581, 123582 • same locality as for preceding; 6 Nov. 1985; TAIF 123670, 123671 • Taichung City, Chiabautai; 9 Sep. 1962; NTUF 001769, 001771, 001775 • same locality as for preceding; 10 Sep. 1962; NTUF 001773 • Taichung City, Kukuan, 14 Mar. 1971; NTUF 001776.

#### Diagnosis.

Forewing vein M < 2.0 times vein M_1+2_, cell cu_1_ value > 2.0, cell m_1_ value > 1.8. Genal processes massive, blunt apically. Male paramere, in profile, with almost straight anterior margin; apex pointed. Distal segment of aedeagus shorter than paramere, apical third inflated, spoon-shaped. Female proctiger truncate apically.

#### Description.

***Adults*** (Figs 1, 5A, B). Coloration. Body color greenish brown (Fig. 1). Newly emerged individuals light green. Antennae yellow with apices of segments 4, 6, and 8 dark brown, and entire segments 9 and 10 black. Compound eyes dark brown. Ocelli orange. Legs brown. Forewing and hindwing transparent.

Structure. Body large, length from anterior head margin to tip of folded forewing 5.4–6.8 mm; covered in long fine setae. Head (Fig. 2A) nearly as wide as thorax, inclined in a 45° angle from longitudinal body axis. Vertex 1.8–2.0 times as wide as long, moderately concave at posterior margin. Genal processes prominent, 0.8–1.0 times as long as vertex along mid-line, divergent, conical, blunt at apex, pubescent. Antenna (Fig. 2B) slender, 10-segmented, 1.5–1.8 times as long as head width, relative length of flagellar segments as 1.0: 0.4: 0.3: 0.4: 0.3: 0.3: 0.2: 0.2, with a single rhinarium on each of segments 4, 6, 8 and 9; longer, pointed terminal seta 1.1 times and shorter, truncate terminal seta 0.2 times as long as segment 10. Thorax weakly arched dorsally. Pronotum deflexed from mesothorax in a 45° angle. Legs slender. Meracanthus well developed, horn-shaped, acute at apex (Fig. 2C); metatibia 0.9–1.2 times as long as head width, slightly inflated basally with four or five small spines, with 1+2 or rarely 1+3 apical spurs. Forewing (Fig. 2D) 5.4–6.4 times as long as head width, 2.5–2.7 times as long as wide, widest slightly distal to the middle; wing apex subacute, lying in cell m_1_ near apex of vein M_1+2_; vein R+M+Cu strictly trifurcating into veins R, M and Cu; vein Rs moderately long, irregularly, concavely curved to fore margin of wing; vein M weakly curved with very long diverging branches; cell m_1_ large; vein Cu_1a_ strongly curved in the basal third; cell cu_1_ smaller than cell m_1_; line connecting apices of veins Rs and Cu_1a_ distal of bifurcation of vein M; surface spinules absent except for base of cell cu_2_; radular spinules present along wing margin in the middle of cells m_1_, m_2_ and cu_1_. Hindwing 0.7 times as long and 0.5 times as wide as forewing; costal margin with five or six setae proximal to costal break, setae distal to costal break clearly divided into two groups. Abdominal tergites glabrous except for a lateral row on either side of tergite 2 in male and tergite 3 in female.

Male terminalia (Fig. 3A–C). Proctiger tubular, in profile broadly convex posteriorly, covered in long setae except for basal third laterally (Fig. 3A). Subgenital plate subglobular, with long setae laterally and ventrally; dorsal margin angular in basal third. Paramere (Fig. 3B) about as long as proctiger; in profile lamellar, irregularly narrowing to apex which is acute and weakly directed anteriad; outer face glabrous except for margins and apex; inner face beset with long setae mostly along fore and hind margins as well as basally. Distal segment of aedeagus (Fig. 3C) shorter than paramere, apical third inflated, spoon-shaped; sclerotized end tube of ductus ejaculatorius short, sinuous. Female terminalia (Fig. 3D) cuneate, short. Proctiger with straight dorsal margin and blunt apex, as long as subgenital plate; with a transverse row of long setae in the middle and long setae apically; circumanal ring one third as long as proctiger, consisting of two unequal rows of pores (Fig. 3E). Subgenital plate, in profile, irregularly triangular, acute at apex; beset in long hairs laterally and ventrally. Dorsal valvulae cuneate, ventral valvulae straight lacking teeth.

Measurements (range, mean ± SD) in mm (5 males, 5 females). Body length (including forewing) ♂ 5.38–6.38, 6.04 ± 0.33; ♀ 6.00–6.81, 6.60 ± 0.27. Head width ♂ 0.83–0.95, 0.89 ± 0.05; ♀ 0.85–0.98, 0.93 ± 0.04. Vertex length ♂ 0.25–0.30, 0.28 ± 0.02; ♀ 0.28–0.30, 0.30 ± 0.01. Genal cone length ♂ 0.23–0.25, 0.25 ± 0.01; ♀ 0.28–0.30, 0.28 ± 0.01. Antenna length ♂ 1.23–1.58, 1.43 ± 0.11; ♀ 1.38–1.55, 1.46 ± 0.07. Metatibia length ♂ 0.88–0.95, 0.93 ± 0.03; ♀ 0.88–0.98, 0.92 ± 0.03. Forewing length ♂ 4.44–5.31, 5.02 ± 0.31; ♀ 5.25–5.88, 5.63 ± 0.19.

***Fifth instar immatures*** (Figs 4A, 5D). Coloration. General color pale green. Body (Fig. 4A) form oval, 1.4–1.5 times as long as wide; sclerotized dorsally, membranous ventrally. Dorsal body surface covered in short normal setae or subacute sectasetae; margin of head (Fig. 4B), forewing (Fig. 4C) and hindwing pads (Fig. 4D), as well as caudal plate (Fig. 4E) with long, very slender, subacute sectasetae which are relatively densely spaced (distance between setae 0.5–1.0 times their length). Antenna (Fig. 4G) weakly curved; 8-segmented; scape and pedicel much thicker than flagellum; relative length of flagellar segments as 1.0: 0.6: 0.3: 0.3: 0.4: 2.3; with a single subapical rhinarium on each of segments 4 and 6, and two on segment 8. Legs moderately long, femur about as long as tibiotarsus; tarsus with two well-developed claws, tarsal arolium (Fig. 4F) longer than claws, triangular, with unguitractor but lacking pedicel. Forewing pad 3.0–3.8 times long as broad, 3.0–3.4 times as long as antenna; humeral lobe relatively short, reaching about basal third of eye, angular. Caudal plate broadly rounded caudally, 0.6–0.7 times as long as wide. Circumanal ring (Fig. 4H) relatively small, transverse, narrowly oval, 0.2–0.3 times as wide as caudal plate; in ventral position close to hind of caudal plate; outer ring composed of 2–5 rows of pores.

Measurements (range, mean ± SD) in mm (5 immatures). Body length 2.63–2.83, 2.76 ± 0.08. Head width 0.85–0.93, 0.88 ± 0.03. Antenna length 0.43–0.48, 0.45 ± 0.02. Metatibiotarsus length 0.60–0.68, 0.65 ± 0.03. Forewing pad length 1.35–1.45, 1.41 ± 0.04. Caudal plate length 0.85–1.00, 0.97 ± 0.07. Caudal plate width 1.45–1.55, 1.52 ± 0.04. Circumanal ring width 0.38–0.44, 0.41 ± 0.02.

#### Etymology.

Named after the Chinese common name of the host plant, 土肉桂, transliterated as “turouguei“; to be treated as a noun in the nominative singular standing in apposition.

#### Distribution.

Taiwan.

#### Host plant and its phenology.

*Cinnamomum osmophloeum* Kaneh. (Lauraceae). Leaf and flower buds of *C. osmophloeum* appear in late April. Young leaves grow from late May to late June and flowers bloom from early June to August. Fruits ripen from September to November.

#### Biology.

*Trioza turouguei* sp. nov. is univoltine and induces pea-shaped galls (Fig. 5C, D) on the stems of new shoots of *C. osmophloeum*. The galls are unilocular with a single immature in each chamber. The annual life cycle of the gall is synchronized with the host phenology and passes through the following four stages of development as defined by Rohfritsch (1992). (1) Initiation: this stage is very short lasting from late April to the early May. After the first instar inserts its stylets into the phloem and injects saliva, the area on which it sits, either a flower, or leaf petiole, or a tender stem, transforms into a tiny pit and the surrounding area starts swelling. (2) Growth and differentiation: from late May to November, the gall forms and completely covers the immature. The second instar appears in late May and lasts until September. The third and fourth instars can be found in October and November, respectively. (3) Maturation: in December, the gall enters the maturation stage, and the immatures attain the final (fifth) instar. The gall reaches its maximum size with a diameter/length of 5.0/7.8 mm. (4) Dehiscence: during January and March, the gall dehisces by mechanical force in the gall tissue. The final instar immatures crawl out of the gall where the adults emerge. Soon after, the adults start mating.

#### Affinities.

Hollis and Martin (1997) listed ten named triozid species from the Old World and one undescribed *Trioza* species from the New World associated with *Cinnamomum* spp. An updated list of the Old World species is provided in Table 1, taking into account taxonomical changes of the last 20 years including some proposed here. Despite a certain morphological resemblance among the Old World species, it is questionable if the group is monophyletic. The species share (mostly) following characters: genal processes developed, more than half vertex length; antennal segment 3 very long (not in *T. hangzhouica* (Li, 1994)); terminal antennal setae strongly unequal in length; forewing transparent, with short concave or sometimes sinuous vein Rs; hindwing over half as long as forewing; metatibia with a group of basal spines and 1+2 small apical spurs (1+3 in *T. exoterica* Yang, 1984 and *T. nigricamphorae* Li, 1993).

Li (2005, 2011) erected two ill-defined, probably polyphyletic genera *Triozopsis* (type species *Trioza nigricamphorae*) and *Metatriozidus* (type species *Metatriozidus ileicisuga* Li, 2011) in which he also placed species associated with *Cinnamomum*. Here we adopt the broad concept of *Trioza* Foerster by Hollis (1984) and consider *Metatriozidus* and *Triozopsis* as subjective synonyms following Yang et al. (2013).

Based on the examination of relevant types (CAUB) we propose here following new synonymy: *Trioza inflata* Li, 1992, = *Trioza xiangicamphorae* Li, 1992, syn. nov.

Mound and Halsey (1978) transferred *Siphonaleyrodes formosanus* Takahashi, 1932 from whiteflies to psyllids and synonymised it with *Trioza cinnamomi* (Boselli, 1931). According to the original description, the immatures of *S. formosanus* are relatively slender and possess several rows of marginal sectasetae (Takahashi 1932). Immatures of *T. cinnamomi* on the other hand are broader and possess only a single row of marginal sectasetae (Miyatake 1969; NHMB data). Based on this evidence, we conclude that the two taxa are not conspecific and remove the former from synonymy with *Siphonaleyrodes formosanus*, stat. rev. The species is currently only known from immatures which makes it difficult to place this genus within the current classification of Triozidae (Burckhardt and Ouvrard 2012, Percy et al. 2018). The type material of *S. formosanus* is apparently lost (M. M. Yang, pers. obs.).

*Trioza turouguei* sp. nov. differs from the other species associated with *Cinnamomum* as indicated in the following keys. In particular, it is diagnosed by details of the male and female terminalia and the multilayered circumanal ring in the immature.

**Table 1.** Old World Triozidae associated with *Cinnamomum* (Lauraceae). Plant names marked with asterisk are confirmed hosts as defined by Burckhardt et al. (2014).

Psylloid species	Host species	Gall type	Distribution	Reference
*Siphonaleyrodes formosanus* Takahashi, 1932, stat. rev.	*Cinnamomum reticulatum Hayata	pit galls	Taiwan	Takahashi (1932)
*Trioza camphorae* Sasaki, 1910	*Cinnamomum camphora (L.) J. Presl	pit galls	India ?, Japan, Taiwan, China	Li (2011), Yang et al. (2013), Burckhardt et al. (2018)
*Trioza camphoricola* Li, 1993	*Cinnamomum camphora* (L.) J. Presl	?	China	Li (2011)
*Trioza cinnamomi* (Boselli, 1931)	*Cinnamomum doederleinii Engl., *C. loureiroi Nees, *C. tenuifolium (Makino) Sugim., *C. yabunikkei H. Ohba, *Neolitsea aciculata* (Blume) Koidz.	pit galls	Japan, Korea (the record from Taiwan is erroneous as *T. cinnamomi* was described from Japan and not Taiwan)	Hodkinson (1983, 1986), Cho et al. (2017)
*Trioza exoterica* Yang, 1984; = *Trioza parthenoxyli* Yang & Li, in Li & Yang, 1985	*Cinnamomum porrectum* (Roxb.) Kosterm., *Cryptocarya chinensis (Hance) Hemsl. (Lauraceae)	leaf curling gall	China, Taiwan	Li (2011)
*Trioza guipicircularis* (Li, 2011) = *Trioza circularis* Li, 1993 nec Froggatt, 1901; primary homonym	*Cinnamomum austrosinense* H. T. Chang	closed gall (not pit gall as recorded in Hollis and Martin 1997)	China	Li (2011)
*Trioza hangzhouica* (Li, 1994)	*Cinnamomum tenuifolium* (Makino) Sugim.	unknown	China	Li (2011)
*Trioza inflata* Li, 1992, = *Trioza xiangicamphorae* Li, 1992, syn. nov.	*Cinnamomum iners* Reinw. ex Blume, *C. verum* J. Presl	?	China	Li (2011)
*Trioza macularicamphorae* Li, 1992	*Cinnamomum iners* Reinw. ex Blume	?	China	Li (2011)
*Trioza magnicamphorae* Li, 1993	*Cinnamomum camphora* (L.) J.Presl	?	China	Li (2011)
*Trioza monri* Burckhardt, 2018 = *Trioza laqueus minor* Kandasamy, 1986	*Cinnamomum* sp.	?	India	Burckhardt et al. (2018)
*Trioza nigricamphorae* Li, 1993	*Cinnamomum camphora* (L.) J.Presl	?	China	Li (2011)
*Trioza turouguei* sp. nov.	*Cinnamomum osmophloeum Kaneh.	mung-pea like stem gall	Taiwan	this paper
*Trioza pseudocinnamomi* Li, 1993	*Cinnamomum burmanni* (Nees & T. Nees) Blume	?	China	Li (2011)

### Keys to the Old World Triozidae associated with *Cinnamomum*

**Adults**

(Adults of *Siphonaleyrodes formosanus* are unknown)

**Table:** 

1	Metatibia with 1+3 apical spurs	**2**
–	Metatibia with mostly 1+2 apical spurs	**3**
2	Genal processes shorter than vertex along midline. Vein Rs of forewing short, concavely curved towards fore margin	***Trioza exoterica* Yang**
–	Genal processes longer than vertex along midline. Vein Rs of forewing long, sinuous	***Trioza nigricamphorae* Li**
3	Forewing vein M > 2.0 times vein M_1+2_	**4**
–	Forewing vein M < 2.0 times vein M_1+2_	**5**
4	Genal processes about as long as vertex along midline. Vein Cu of forewing longer than Cu_1b_. China	***Trioza hangzhouica* (Li)**
–	Genal processes distinctly shorter than vertex along midline. Vein Cu of forewing shorter than Cu_1b_. India	***Trioza monri* Burckhardt**
5	Forewing with cell cu_1_ value > 2.0	**6**
–	Forewing with cell cu_1_ value < 1.9	**9**
6	Genal processes slender, subacute apically. Forewing with cell m_1_ value < 1.8	**7**
–	Genal processes massive, blunt apically. Forewing with cell m_1_ value > 1.8	**8**
7	Forewing widest in apical third; apex subacute	***Trioza cinnamomi* (Boselli)**
–	Forewing widest in the middle; apex narrowly rounded	***Trioza macularicamphorae* Li**
8	Paramere, in profile, with basal lobe anteriorly; apex blunt. Female proctiger with digitiform apical process	***Trioza magnicamphorae* Li**
–	Paramere, in profile, with almost straight anterior margin; apex pointed. Female proctiger truncate apically	***Trioza turouguei* sp. nov.**
9	Genal processes around two thirds of vertex length measured along midline	***Trioza camphoricola* Li**
–	Genal processes as long as or longer than vertex along midline	**10**
10	Male proctiger short, weakly produced posteriorly, without very long conspicuous setae along hind margin. Paramere, in profile, distinctly narrowed in apical third	**11**
–	Male proctiger long, strongly produced posteriorly, with long conspicuous setae along hind margin. Paramere, in profile, not strongly narrowed in apical third.	**12**
11	Forewing narrowly rounded apically; vein Rs of forewing relatively long, almost straight, slightly turned towards fore margin apically	***Trioza camphorae* Sasaki**
–	Forewing pointed apically; vein Rs short, concavely curved towards fore margin	***Trioza inflata* Li**
12	Forewing 2.3 times as long as broad. Paramere, in profile, lamellar, truncate apically	***Trioza guipicircularis* Li**
–	Forewing 2.9 times as long as broad. Paramere, in profile, lanceolate, subacute apically	***Trioza pseudocinnamomi* Li**

**Immatures**

(Immatures of *Trioza camphoricola*, *T. guipicircularis*, *T. hangzhouica*, *T. inflata*, *T. macularicamphorae*, *T. magnicamphorae*, *T. monri*, *T. nigricamphorae* and *T. pseudocinnamomi* are unknown)

**Table:** 

1	Body relatively slender, > 1.6 times as long as wide	**2**
–	Body relatively broad, < 1.5 times as long as wide	**3**
2	Body margin with several rows of sectasetae………..	***Siphonaleyrodes formosanus* Takahashi**
–	Body margin with a single row of sectasetae	***Trioza exoterica* Yang**
3	Outer circumanal ring consisting of 2–5 rows of pores	***Trioza turouguei* sp. nov.**
–	Outer circumanal ring consisting of a single row of pores	**4**
4	Dorsal outline subcircular, 1.1 times as long as wide. Tarsal arolium circular	***Trioza camphorae* Sasaki**
–	Dorsal outline broadly oval, 1.2 times as long as wide. Tarsal arolium triangular	***Trioza cinnamomi* (Boselli)**

## Discussion and conclusions

Hollis and Martin (1997) showed that sap sucking insects colonized Lauraceae more successfully than chewing insects by an order of magnitude of percentage of number of species associated with this family. The reason may be the phytochemicals in the leaves deterring chewing insects more efficiently than sucking insects. Among psyllid host plant families, Lauraceae is ranked 7^th^ in terms of number of associated psyllid genera (Ouvrard et al. 2015). More than two thirds of the psyllid species associated with Lauraceae belong to the Triozidae and of these almost two thirds induce galls or other deformations on their hosts (Hollis and Martin 1997). The Old World triozids developing on *Cinnamomum* (Table 1) fit this pattern. Of the 14 species, five with confirmed hosts (hosts marked with asterisk in Table 1) induce galls. Of the remainder, the association with *Cinnamomum* of seven species is likely but that of two (*Trioza exoterica*, *T. hangzhouica*) is questionable. The former develops on *Cryptocarya chinensis* (host confirmed by the presence of immatures) and *Cinnamomum porrectum* may be just casual plant (Burckhardt et al. 2014). The same is true for *T. hangzhouica*. Among the five species with confirmed hosts, all are monophagous except for *T. cinnamomi* which is narrowly oligophagous.

*Trioza turouguei* sp. nov. is characterized by the induction of pea-shaped galls on stems of its host. Stem galls induced by psyllids are much rarer than those on leaves (Hodkinson 1984, Burckhardt 2005, Yang et al. 2006, Yang and Raman 2007). Examples are *Pachypsylloides* species (Liviidae) on *Calligonum* species (Polygonaceae), two *Pachypsylla* species (Aphalaridae) on *Celtis* species (Cannabaceae), *Egeirotrioza bifurcata* (Mathur 1975) and *Egeirotrioza populi* (Horváth 1915) (Triozidae) on *Populus* species (Salicaceae) or *Calophya rubra* (Blanchard 1852) (Calophyidae) on *Schinus polygama* (Anacardiaceae) (Yang and Mitter 1994, Burckhardt and Basset 2000, Hodkinson 2009).

*Cinnamomum osmophloeum*, the host of *Trioza turouguei* sp. nov., has a scattered distribution in Taiwan growing in broad-leaved forests. Its conservation status is “Vulnerable” in the International Union for Conservation of Nature (IUCN) Red List (Pan 1998). This plant species is not hard to find at low to medium mountain areas in Taiwan, but the majority of trees are planted and only a few grow naturally. The unintentional introduction of *Cinnamomum burmannii* into Taiwan may endanger the natural population of *C. osmophloeum* (Tseng et al. 2008). Contrary to its host, adults, and galls of *Trioza turouguei* were found only in central Taiwan. Adults of *Trioza turouguei* are quite big and the galls are conspicuous on a plant species of medicinal interest. It is, therefore, surprising that the species has not been described before. One reason for this may be the very short period of adult emergence (1–2 weeks). Another reason is certainly that psyllid diversity in general, and that of the tropics in particular, is still insufficiently known.

## Supplementary Material

XML Treatment for Trioza turouguei
